# Chronic Alcoholism and Its Health Implications: A Case Report

**DOI:** 10.7759/cureus.40364

**Published:** 2023-06-13

**Authors:** Amirmohsen Arbabi, Bella Garg, Sridhar M Reddy, Paryus Patel

**Affiliations:** 1 Internal Medicine, Centinela Hospital, Los Angeles, USA; 2 Interventional Cardiology, Centinela Hospital, Los Angeles, USA; 3 Critical Care Medicine, Centinela Hospital, Los Angeles, USA

**Keywords:** cirrhotic cardiomyopathy, alcohol-associated cirrhosis, dilated cardiomyopathy, atrial fibrillation with rapid ventricular response, alcohol use disorder

## Abstract

Individuals with chronic alcohol use can be asymptomatic for a prolonged period of time and then exhibit signs of advanced heart and liver diseases with an abrupt onset. Herein, we present a case of a 60-year-old male with severe alcohol use disorder who presented with newly diagnosed atrial fibrillation (AF) with rapid ventricular response (RVR), dilated cardiomyopathy (DCM), and alcohol-associated cirrhosis following an episode of binge drinking.

## Introduction

As the third leading preventable cause of death in the United States, excessive alcohol use accounts for more than 85,000 deaths per year [1-2]. Presently, alcohol is believed to be toxic to cardiac myocytes through increased oxygen free radical production and defects in cardiac protein synthesis [3]. Consequentially, this may lead to major cardiovascular complications such as dilated cardiomyopathy (DCM) and arrhythmias including atrial fibrillation (AF) with rapid ventricular response (RVR) [3]. Chronic alcohol use also increases the chance of broad-spectrum hepatic disorders such as steatosis, steatohepatitis, and, ultimately, cirrhosis from oxidative stress [4]. This case report aims to spread awareness about the importance of screening and early detection with reduction or cessation of alcohol intake in high-risk patients in order to prevent or even possibly reverse advanced liver and heart diseases.

## Case presentation

**Figure 1:**
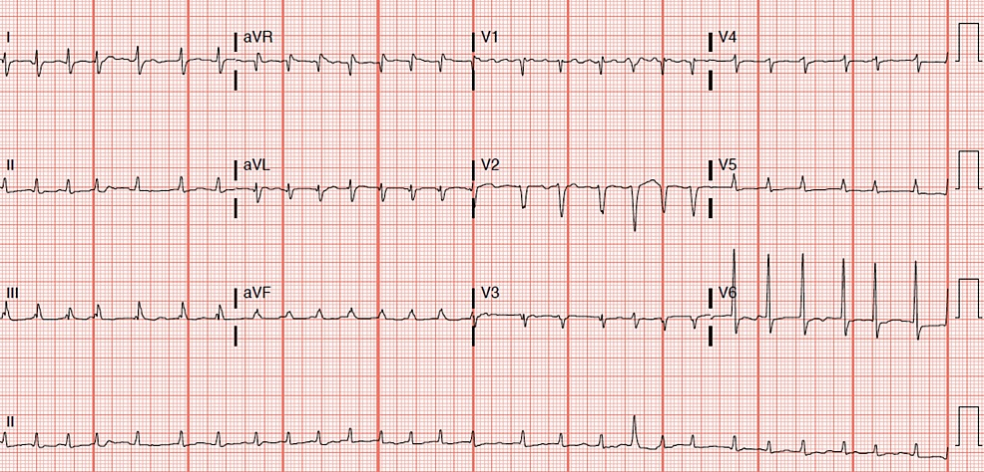
AF with RVR. AF, atrial fibrillation; RVR, rapid ventricular response

A 60-year-old male with a past medical history of severe alcohol use disorder presented to the emergency room (ER) with a chief complaint of a racing heart and palpitations. He stated he had drunk six glasses of cognac the night before his symptoms arose. While walking the next day, he experienced a sudden onset of dizziness and palpitations with mild shortness of breath. He denied any symptoms of subjective fever, altered mental status, visual disturbances, chest pain, cough, nausea, emesis, or abdominal pain. Additionally, he complained of orthopnea, paroxysmal nocturnal dyspnea, abdominal distention, and bilateral leg swellings for the past few months prior to admission. He admitted that he usually drinks three glasses of hard liquor daily for 30+ years, however, he recently went “partying” and thus, was drinking excessively. He refuted any cigarette or illicit drug use. On admission, he was tachycardic with a heart rate of 160 and his electrocardiogram (EKG) showed AF with RVR (Figure 1). This temporarily reverted to normal sinus rhythm after he was given diltiazem in the ER. The physical examination revealed moderate ascites and 2+ pitting edema bilaterally in his lower extremities. Lab abnormalities included macrocytosis, mild transaminitis, hyperbilirubinemia of 2.1 mg/dL, hypoalbuminemia of 2.4 g/dL, and an increased international normalized ratio (INR) of 1.5. His urine toxicology result was negative, and levels of salicylate, acetaminophen, and blood ethanol were normal. The N-terminal pro-brain natriuretic peptide (NT-proBNP) level was elevated at 11,000 pg/mL and cardiomegaly was observed on his chest X-ray (CXR). Based on these findings, both cardiology and gastrointestinal (GI) services were consulted. His echocardiogram revealed a severely dilated left ventricle, severe systolic dysfunction with an ejection fraction of 15%-20%, and moderate pulmonary hypertension with a right ventricular systolic pressure (RVSP) of 45-50 mmHg. The CT scan showed cirrhotic liver with moderate ascites, anasarca, evidence of portal hypertension, and chronic pancreatitis with a 5 cm x 7 cm pancreatic tail loculated pseudocyst. The alpha-fetoprotein (AFP) and carcinoembryonic antigen (CEA) markers were both normal. Paracentesis was performed and 1200 mL of yellow-colored fluid was aspirated. The ascitic fluid analysis showed a total protein of 2.8 g/dL, albumin of 1.3 g/dL with a serum-ascites albumin gradient (SAAG) of 1.1, white blood cell (WBC) count of 103 /cu mm, and neutrophils of 6%. Another episode of AF with RVR occurred and he was subsequently placed on metoprolol and digoxin. Due to his cirrhosis and coagulopathy, he was not a good candidate for amiodarone and anticoagulation. Other medications included furosemide and spironolactone. After paracentesis, the patient’s clinical status improved significantly, and he was appropriately counseled on alcohol use cessation. Once his clinical condition was stable, he was discharged from the hospital and advised to follow up as an outpatient with GI and cardiology for an upper endoscopy and ischemic workup, respectively.

## Discussion

This case report discusses a 60-year-old male with severe alcohol use disorder who presented with a newly diagnosed AF with RVR, DCM, and cirrhosis. Our case demonstrates the consequential health risks of chronic alcoholism and highlights the significance of timely screening and early intervention for at-risk alcohol use. DCM is characterized by dilation and impaired contraction of one or both ventricles and is caused by a variety of disorders such as genetic mutations, infections, autoimmune disorders, toxins and chemicals, pregnancy, endocrinologic disorders, deposition diseases, nutritional deficiencies, and neuromuscular diseases [5-7]. Persistent alcohol overindulgence is the leading cause of an acquired or secondary DCM [8-9]. Different mechanisms including defects in mitochondrial function, oxidative stress, and apoptosis appear to play a role in the development of alcohol-induced cardiomyopathy [10]. This case portrays alcohol-induced cardiomyopathy as the recognized diagnosis based on his history of chronic excessive alcohol consumption. Nevertheless, his presentation with AF with RVR can raise suspicion for arrhythmia-induced cardiomyopathy based on his recent history of binge drinking. Another possibility is cirrhotic cardiomyopathy independent of alcohol exposure with evidence supported by his CT scan which showed a cirrhotic liver [11]. Acute cardiac arrhythmias like AF that occur following excessive alcohol intake during weekends or holidays suggest a condition known as “holiday heart syndrome" [12]. In this case, AF with RVR is most likely related to alcohol-induced cardiomyopathy. However, holiday heart syndrome should be considered as a possible differential for his AF based on the timing of his recent binge drinking and the successive AF that occurred [13].

## Conclusions

In the context of alcohol-induced cardiomyopathy and cirrhosis, early detection with reduction or cessation of alcohol intake correlates with an improvement in cardiac and liver functions. Therefore, we recommend that primary care physicians screen all adults 18 and older for unhealthy alcohol use. This information can help healthcare providers educate their patients on the benefits of alcohol reduction/cessation and its positive impact on better health and well-being.
